# Effect of Temperature and Growth Time on Vertically Aligned ZnO Nanorods by Simplified Hydrothermal Technique for Photoelectrochemical Cells

**DOI:** 10.3390/ma11050704

**Published:** 2018-04-29

**Authors:** Laimy Mohd Fudzi, Zulkarnain Zainal, Hong Ngee Lim, Sook-Keng Chang, Araa Mebdir Holi, Mahanim Sarif@Mohd Ali

**Affiliations:** 1 Department of Chemistry, Faculty of Science, Universiti Putra Malaysia, 43400 UPM Serdang, Selangor, Malaysia; laimy93@yahoo.com (L.M.F.); janetlimhn@gmail.com (H.N.L.); skchang28@hotmail.com (S.-K.C.); 2 Materials Synthesis and Characterization Laboratory, Institute of Advanced Technology, Universiti Putra Malaysia, 43400 UPM Serdang, Selangor, Malaysia; mahanimsarif@gmail.com; 3 Department of Physics, College of Education, University of Al-Qadisiyah, Al-Diwaniyah, Al-Qadisiyah 58002, Iraq; araa.holi@qu.edu.iq; 4 Forest Product Division, Forest Research Institute Malaysia, 52109 Kepong, Selangor, Malaysia

**Keywords:** hydrothermal, dip coating, ZnO, nanorods, photoelectrochemical

## Abstract

Despite its large band gap, ZnO has wide applicability in many fields ranging from gas sensors to solar cells. ZnO was chosen over other materials because of its large exciton binding energy (60 meV) and its stability to high-energy radiation. In this study, ZnO nanorods were deposited on ITO glass via a simple dip coating followed by a hydrothermal growth. The morphological, structural and compositional characteristics of the prepared films were analyzed using X-ray diffractometry (XRD), field emission scanning electron microscopy (FESEM), and ultraviolet-visible spectroscopy (UV-Vis). Photoelectrochemical conversion efficiencies were evaluated via photocurrent measurements under calibrated halogen lamp illumination. Thin film prepared at 120 °C for 4 h of hydrothermal treatment possessed a hexagonal wurtzite structure with the crystallite size of 19.2 nm. The average diameter of the ZnO nanorods was 37.7 nm and the thickness was found to be 2680.2 nm. According to FESEM images, as the hydrothermal growth temperature increases, the nanorod diameter become smaller. Moreover, the thickness of the nanorods increase with the growth time. Therefore, the sample prepared at 120 °C for 4 h displayed an impressive photoresponse by achieving high current density of 0.1944 mA/cm^2^.

## 1. Introduction

The wide-band gap semiconductor, ZnO with a band gap value of 3.37 eV, belongs to the II–IV group, which is widely used in optoelectronic applications due to its high electron mobility [1]. Besides ZnO, there are various types of metal oxides being applied in photoelectrochemical, such as Fe_2_O_3_, Nb_2_O_5_, CeO_2_, TiO_2_ and so on [2]. ZnO nanostructures exist in different forms, such as nanobelts [3], nanorings [4], nanohelices [5], nanorods [6], nanocombs [7] and a tetrapod [8] governed by its synthesis parameters [9]. For instance, in a gas sensor, due to its sensitivity towards various gases [10], it can act as a highly conductive field-effect transistor and can be manipulated through annealing temperature [11], thermoelectric performance enhancer [12], solar-blind ultra-violet detectors [13], solar cells [14] and so on. Thus, ZnO thin films may play an important role in the development of advanced materials.

Up till this day, numerous methods have been discovered for the deposition of ZnO thin films [15]. Besides dip coating, techniques like spin coating [16], chemical bath deposition [17], chemical vapor deposition [18], atomic layer deposition [19,20], sputtering [21,22], and successive ion layer adsorption and reaction (SILAR) [23] have been explored. Although, the spin coating method produces uniform thin films, it is difficult to apply on large substrates as a high spin rate should not be applied. Moreover, a typical spin coating treatment utilizes merely 2–5% of the precursor to be dispensed onto the substrate, leaving the remaining 95–98% of the precursor to be wasted by splashing off into the coating bowl [24]. Despite the fact that the chemical bath deposition is a relatively simple technique, it has the ability to fabricate thin films with good reproducibility. However, it is unable to deposit a few layers of materials and furthermore, repeating the coating will increase the risk of peeling off the deposited films [25]. Chemical vapor deposition is favored by many researchers in the fabrication of thin films as this technique is able to be applied on different types of materials with a high purity deposition. This may be due to the distillation process, where impurities are removed from the gaseous precursors. However, precursors used for chemical vapor deposition can be highly toxic, corrosive, or even explosive. Not only does this technique require quite pricey precursors, but one of its major drawbacks is the depositions are conducted at extremely high temperatures and this may lead to stresses between the deposited thin film and the substrate due to different thermal expansion coefficients, which will ultimately cause the deposited thin film to be mechanically unstable [26]. Atomic layer deposition may have a disadvantage as this technique provides a very low deposition growth rate in which only a few angstroms thick of monolayer is deposited during each cycle and it may take several hours to produce a few microns thickness of deposition. Moreover, atomic layer deposition is very sensitive to impurities as a high degree of cleanliness is essential and the existence of any impurities from the chemicals used or even a gas precursor may lead to the growth of very poor quality films [27]. In the sputtering process, ZnO thin films that were produced were not preferable for high temperature applications, as these films possess electrical resistivity that increases from three to ten orders of magnitude due to the chemisorption of oxygen [28]. Although SILAR is a relatively direct and simple technique, the applied procedures are tedious and time consuming [29]. Therefore, dip coating would be a good alternative for the formation of large area nanorod arrays for various optoelectronic applications due to its ability to produce homogeneous films. Additionally, dip coating does not require any expensive or complicated instruments or machinery to achieve a good deposition of thin films. Moreover, numerous research studies have reported on the fabrication of ZnO nanorods using a combination of dip coating and hydrothermal growth methods. In this work, a simple, low cost set-up with sealed glass vials was used instead of an expensive autoclave system, which is typically applied in traditional hydrothermal growth treatment [6]. Thus, ZnO seed layers were deposited onto ITO glass, followed by a hydrothermal growth treatment to enable the seeds to grow into nanorods and the effects of the temperature and the time of hydrothermal growth treatments were investigated.

## 2. Materials and Methods

### 2.1. Preparation of ZnO Nanorods

ZnO nanorods were synthesized by a sol-gel dip coating technique followed by a hydrothermal growth treatment. ITO glass substrates were sonicated in acetone, 2-propanol, and deionized water to remove impurities as well as to activate the surface. The precursor solution was prepared by mixing 0.2 M of zinc acetate dehydrate in ethanol solution with 0.2 M diethanolamine under the condition of 60 °C for 30 min followed by aging overnight. Then, the prepared precursor solution was coated onto ITO glass via the dip coating technique for 40 s and then the substrate was heated at 100 °C to eliminate the remaining solvent. A total of three coating layers were deposited to ensure a dense and uniform dispersion of ZnO seed layer was deposited. Then, the coated substrates were annealed at 350 °C for 1 h. Substrates that were coated with ZnO seed layers were treated with hydrothermal growth treatment in order to obtain the ZnO nanorods. A mixture of 0.04 M zinc nitrate 6-hydrate and 0.04 M hexamethylenetetramine was prepared and the annealed samples were then placed in the vials that were filled with the prepared mixture, followed by placing them into an oil bath with the temperature set at 90 °C, 100 °C, 110 °C, 120 °C, and 130 °C for 4 h. The heated samples were then taken out from the vials and rinsed with deionized water and left to dry. The optimum hydrothermal growth temperature and time (first, second, third, and fourth hour) was determined based on the obtained structural and optical characteristics, as well as the photoelectrochemical performance shown by the tested thin films.

### 2.2. Characterization of ZnO Nanorods

The morphologies and elemental analyses of the prepared thin films were obtained from a field emission scanning electron microscopy (FESEM, JEOL Ltd., Tokyo, Japan) using a JSM-7600F equipped with an INCA energy dispersive X-ray spectrometer (EDX, JEOL Ltd., Akishima, Tokyo, Japan) and operated at 10 kV. Meanwhile the structural properties of the prepared samples were analyzed by using a X-ray diffractometer (X’Pert PRO Panalytical Philips, Almelo, Netherlands) that employs a Cu-Kα anode at the range of 20 < θ < 80 with PanalyticalX’Pert Pro MPD software. The absorbance spectra of the prepared samples were measured using a Lambda 20 ultraviolet-visible spectrophotometer (Perkin Elmer Instruments, Waltham, MA, United States). Ultraviolet-visible Spectroscopy (UV-Vis, Perkin Elmer Instruments, Waltham, MA, United States) is the most common technique used to measure the absorption of the sample by exposing it to a light source with the wavelength ranging from 800 to 200 nm. Based on the absorption spectrum data obtained, band gap energy of samples can be calculated via a Tauc plot [30] as follows:

(1) $$\left(\alpha \;hv\right)=A{\left(hv-E_{g}\right)}^{p}$$

where α is the absorption coefficient; *hv* is the energy of incident photons; *A* is the function of the refractive index of material, reduced mass, and speed of light; while *E_g_* is the energy band gap of the semiconductor, the value of *p* is ½ since ZnO is a direct band gap material [31] and the band gap energy can be identified via the interception of the straight line obtained from the plotting graph of (*α hv)^2^* vs. *hv.*

### 2.3. Photoelectrochemical Performance of ZnO Nanorods

ZnO nanorods coated onto ITO substrates were used as the working electrode, platinum wire as the counter electrode and Ag/AgCl as the reference electrode in a three electrode system. The photoelectrochemical measurement was carried out using linear sweep voltammetry (Autolab PGSTAT204/FRA32M module, Metrohm AG, Herisau, Switzerland) to control the potential and record the corresponding photocurrent at the scan rate of 20 mV·s^−1^ in a mixture of Na_2_S and Na_2_SO_3_ electrolytes (pH of 13) under the illumination of a halogen lamp that imitates sunlight. The light intensity was measured using a fiber optic spectrometer (Avaspec-2048, Avantes, Apeldoorn, Netherlands) and the obtained value was 100 mW·cm^−2^. The tested samples were illuminated by intermittent or chopping the light source on-and-off every 2 s from a distance of 15 cm between the three electrode system setup and halogen lamp to obtain a comb-like voltammogram in the photoelectrochemical measurement.

## 3. Results and Discussion

### 3.1. Structural and Optical Characterization of ZnO Nanorods

**Figure 1:**
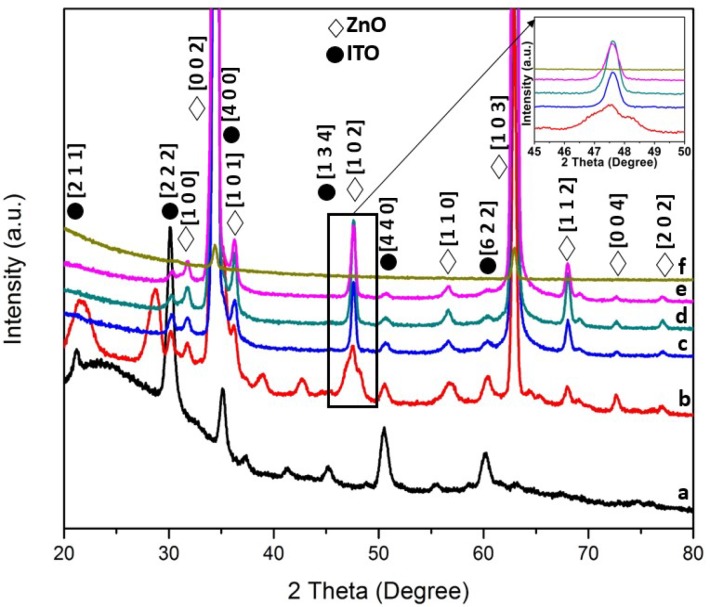
X-ray diffractograms of ZnO/ITO at various hydrothermal growth temperatures for 4 h: (**a**) Blank indium tin oxide substrate; (**b**) 90 °C; (**c**) 100 °C; (**d**) 110 °C; (**e**) 120 °C and (**f**) 130 °C. The inset of Figure 1 represents the (1 0 2) peak plotted from 45° to 50°.

**Figure 2:**
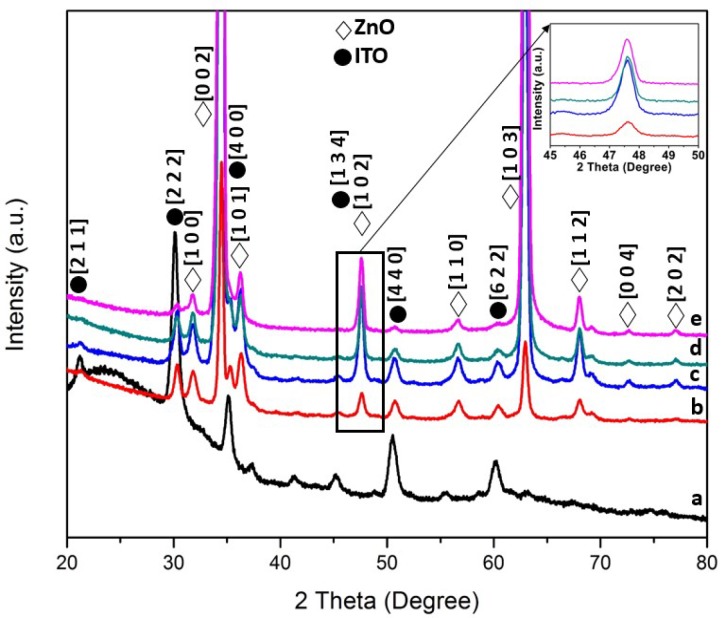
X-ray diffractograms (XRD) of ZnO/ITO at various hydrothermal growth time at 120 °C: (**a**) Blank indium tin oxide substrate; (**b**) 1 h; (**c**) 2 h; (**d**) 3 h and (**e**) 4 h. The inset of Figure 2 represents the (1 0 2) peak plotted from 45° to 50°.

Based on the X-ray diffractograms (XRD, X’Pert PRO Panalytical Philips, Almelo, Netherlands) of ZnO nanorods as shown in Figure 1 and Figure 2, a hexagonal wurtzite structure of ZnO (JCPDS: 00-003-0888) can be represented by (1 0 0), (0 0 2), (1 0 1), (1 0 2), (1 1 0), (1 0 3), (1 1 2), (0 0 4) and (2 0 2) planes. Besides ZnO peaks, tin oxide peaks were also found in the diffractograms as contributed by the substrates used (JCPDS: 01-089-4598). According to the observed XRD patterns shown in Figure 1 and inset of Figure 1 (temperature varied from 90 to 130 °C), it was found that as the hydrothermal growth temperature increases, the intensity of the ZnO peaks increases gradually until 110 °C before showing a decrement from 110 to 130 °C. This phenomenon was clearly observed in the sample prepared at 130 °C in which most of the ZnO peaks were drastically diminished, leaving only peaks on (0 0 2) and (1 0 3) planes. This may lead to the interpretation that the precursor solution of Zn^2+^ used in the hydrothermal process to grow ZnO seeds into nanorods has evaporated as the boiling point of the zinc solution was 125 °C. Thus, the growth of the ZnO seeds was inhibited. Moreover, for Figure 2 and inset of Figure 2, a similar trend could be observed, in which the intensity of the peaks increases as growth time increases from 1 h to 2 h, followed by a decrement from 3 h to 4 h. The Debye–Scherrer formula that was used to calculate the average crystallite size was as follows:

D = 0.94*λ*/*β*cos θ (2)

where *λ* is the X-ray wavelength, *β* is the line broadening at half the maximum intensity (FWHM) and θ is the Bragg angle. For the different temperature parameters, the crystallite size of the ZnO at 90, 100, 110, 120, and 130 °C, was 19.2, 22.3, 26.4, 19.2, and 21.1 nm, respectively, by using a diffraction peak at 2θ = 34.4° on the (0 0 2) plane. On the other hand, the calculated crystallite size values of the ZnO samples prepared at the hydrothermal growth temperature of 120 °C at various duration (1 h, 2 h, 3 h and 4 h) were found to be 24.9, 22.3, 19.2, and 19.2 nm, respectively, by using the same (0 0 2) plane and diffraction peak (2θ = 34.4°). No significant changes were observed when the ZnO and ITO peaks were present as growth time increased. In short, the crystallite size values increased from 90 °C to 110 °C, followed by a decrement at 120 °C before it increased again at 130 °C. As for the growth time parameter, the crystallite size decreases as the growth time increase.

**Figure 3:**
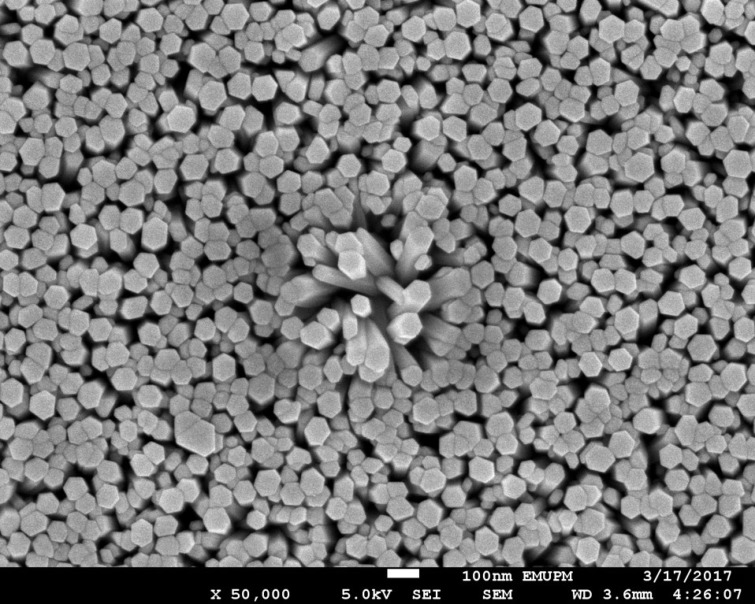
Field emission scanning electron microscopy (FESEM) image of ZnO nanorods prepared at 90 °C with a growth time of 4 h at the magnification of ×50,000.

**Figure 4:**
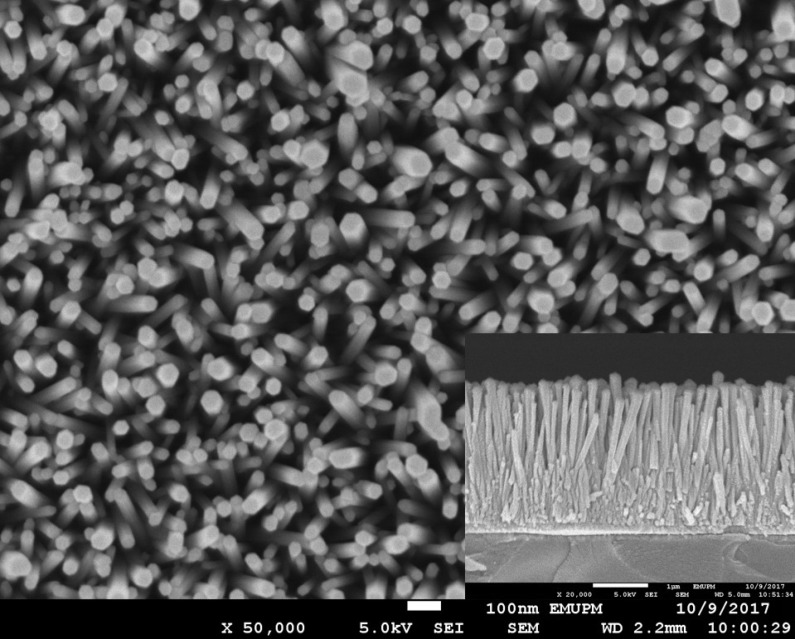
FESEM image of ZnO nanorods prepared at 120 °C with a growth time of 4 h at the magnification of ×50,000. A cross-section of ZnO nanorods prepared under the same conditions is shown in the inset of the figure at the magnification of ×50,000.

FESEM micrographs of the prepared samples underwent 4 h of hydrothermal growth treatment at 90 °C and 120 °C and are depicted in Figure 3 and Figure 4, respectively. The cross-section of the sample prepared at a higher temperature (120 °C) for 4 h was illustrated in the inset of Figure 4. Distinctive rod-like structures of ZnO nanorods were displayed on both micrographs and the vertically aligned nanorods were further proven by observing the side view of the particular thin film (inset of Figure 4). However, when comparing both images, ZnO nanorods in Figure 3 appeared to be very dense and compact, unlike the ZnO nanorods displayed in Figure 4, which have quite spacious surroundings in between each nanorod. In terms of shape, the nanorods observed in Figure 3 have a consistent hexagonal-like shape while the nanorods in Figure 4 were non-uniform and not well-aligned. The values of diameter and thickness of the ZnO nanorods were measured by using ImageJ software and the average values were calculated. The average diameter of the nanostructured ZnO nanorods prepared at 90 °C and 120 °C were 65.1 nm and 37.7 nm, respectively, with a thickness of 2680.2 nm. According to Amin et al. [32], by increasing the growth time, the length of the nanorods will increase, but the density of the nanorods was found to decrease due to the coalescence and fast growth of the nanorods towards the c-axis direction. Consequently, this leads to the interpretation that a larger surface area causes a greater amount of light penetration that contributes to better photoefficiency.

**Figure 5:**
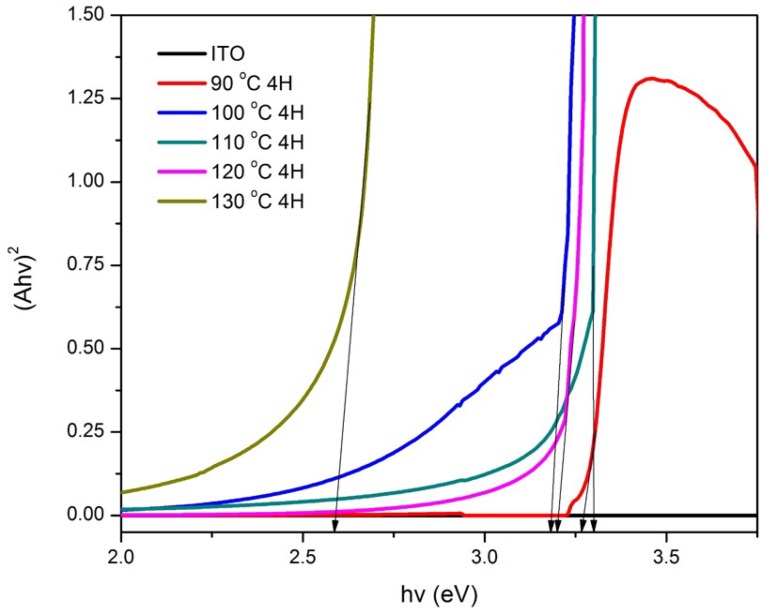
Band gap curves of ZnO/ITO prepared at various hydrothermal temperatures for 4 h at 90 °C, 100 °C, 110 °C, 120 °C, and 130 °C.

**Figure 6:**
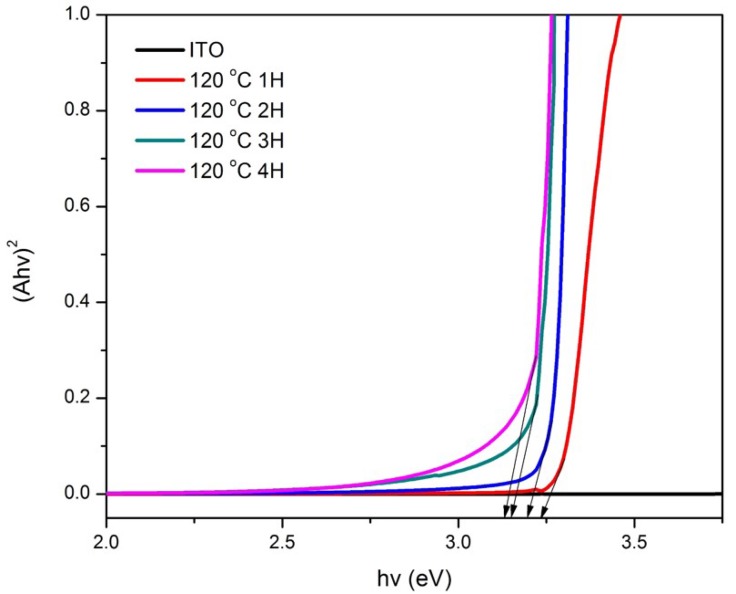
Band gap curves of ZnO/ITO prepared in various growth time at the hydrothermal temperature of 120 °C for 1 h, 2 h, 3 h, and 4 h.

Band gap energy spectra for samples prepared at various hydrothermal temperatures and growth time are represented by Figure 5 and Figure 6, in which the band gap energy was determined via the intersection of the tangent line of the spectra with the *x*-axis [6]. Based on Figure 5, the band gap energy values for the ZnO nanorods prepared at 90, 100, 110, 120, and 130 °C were 3.27, 3.18, 3.30, 3.19, and 2.59 eV, respectively. It was found that the band gap energy values of the samples prepared at the temperatures ranging from 90 to 120 °C were similar to the standard ZnO [33], which was reported at the value of 3.37 eV except for the ZnO prepared at 130 °C. Such results could be explained by the unusual XRD diffractogram that showed the presence of ZnO on the sample, however, it did not optically perform as ZnO according to its band gap energy spectrum, as seen in Figure 5. On the other hand, an obvious trend is shown in Figure 6, in which the band gap energy decreases as the growth time increases. The band gap energy values of the ZnO nanorods prepared at the hydrothermal temperature of 120 °C for 1 h, 2 h, 3 h, and 4 h growth time were 3.24, 3.20, 3.15, and 3.13 eV, respectively. Unlike the temperature factor, even though the band gap energy decreases as growth time increases, the values were still close to the standard ZnO band gap energy (3.37 eV). Thus, this may conclude that 120 °C is the optimum temperature in synthesizing ZnO nanorods as the growth time parameter was not affected significantly.

### 3.2. Photoelectrochemical Measurements of ZnO Nanorods

**Figure 7:**
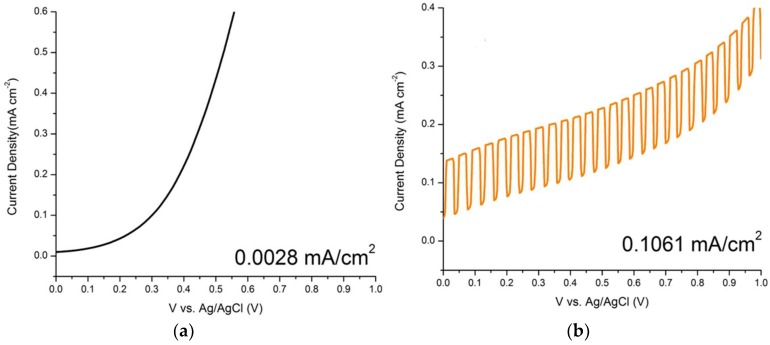

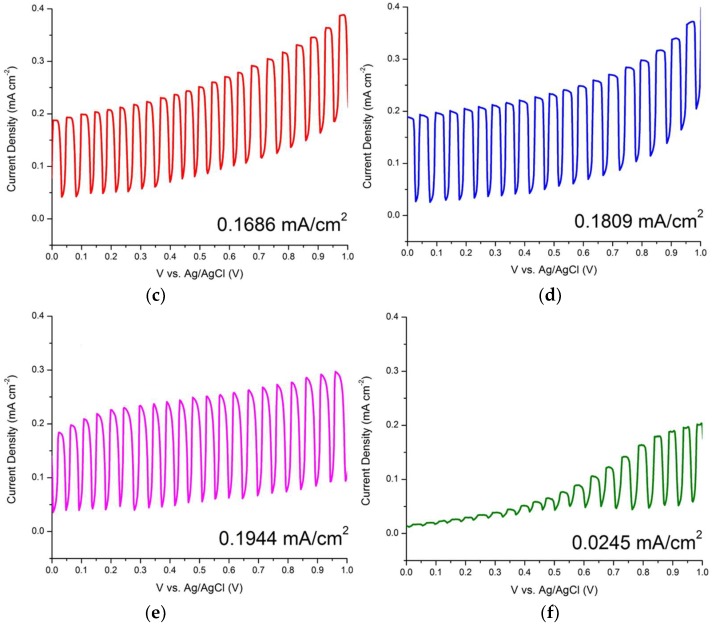
Linear sweep voltammograms of ZnO/ITO prepared at various hydrothermal temperatures in 4 h at the scan rate of 20 mV/s from 0.0 V to +1.0 V: (**a**) Blank indium tin oxide substrate; (**b**) 90 °C; (**c**) 100 °C; (**d**) 110 °C; (**e**) 120 °C and (**f**) 130 °C.

**Figure 8:**
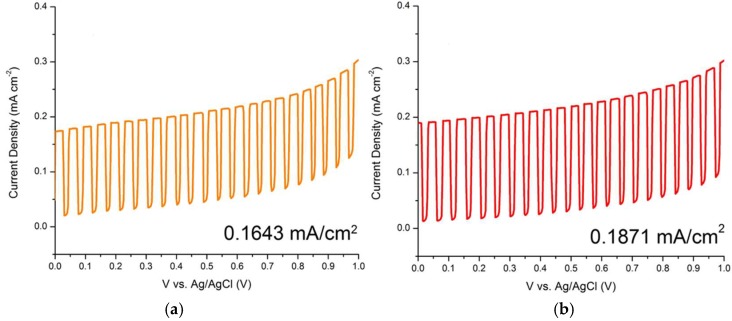

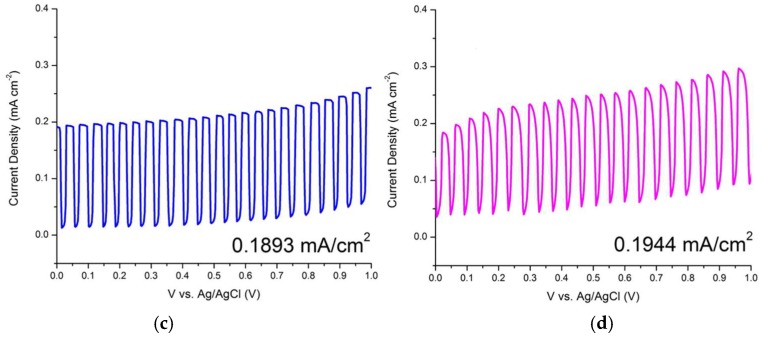
Linear sweep photovoltammograms of ZnO/ITO prepared in various time at the hydrothermal temperature of 120 °C at the scan rate of 20 mV/s from 0.0 V to +1.0 V: (**a**) 1 h; (**b**) 2 h; (**c**) 3 h and (**d**) 4 h.

**Figure 9:**
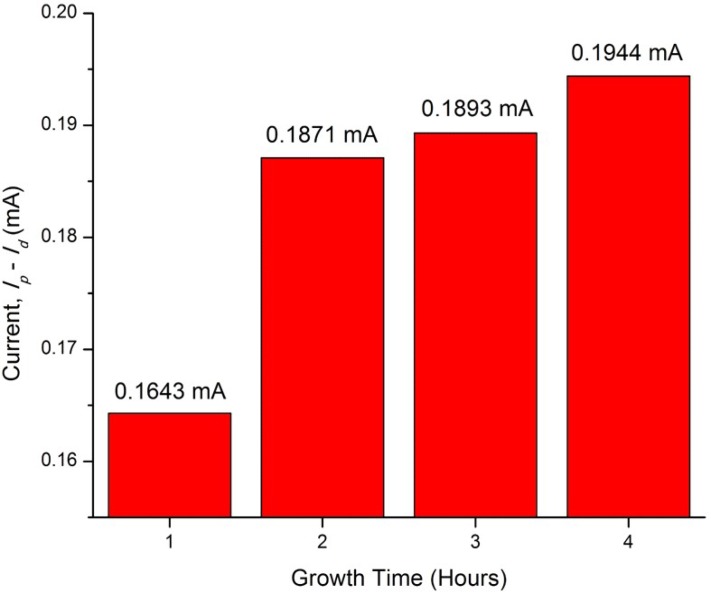
Graph of current *I_p_–I_d_* (mA) plotted against growth time (hours) at potential of +0.5 V.

In order to obtain the optimum condition for the ZnO nanorods growth on the ITO, parameters such as hydrothermal temperature and time have been varied. Based on Figure 7 and Figure 8, the comb-like voltammograms were obtained as a result of intermittent halogen illumination (chopping method) during the photoelectrochemical measurements. According to Figure 7 and Table 1, an increasing trend in current density was observed as the temperature increases from 90 to 120 °C, but the photocurrent density decreased drastically by 87.40% from 120 °C (0.1944 mA/cm^2^) to 130 °C (0.0245 mA/cm^2^). This finding was related to the ZnO nanorods prepared at 130 °C that had an abnormal XRD diffractogram and band gap energy spectrum, along with the explanation deduced above in which at a high temperature of 130 °C, the Zn^2+^ source (crucial precursor for nanorod growth) from the zinc nitrate was vaporized. This inhibited the growth of the ZnO seed layers into nanorods and ultimately hindered the generation of the photocurrent. Additionally, the photocurrent density of the ZnO nanorods prepared at 120 °C (0.1944 mA/cm^2^) was almost two times greater than that of those prepared at 90 °C (0.1061 mA/cm^2^). This finding is in agreement with the UV-Vis and FESEM analyses, whereby the nanorods were so closely packed together that there were hardly any spaces in between the rods, as shown in Figure 3. By observing Figure 4, the difference becomes evident, as there are quite a lot of spaces in between the nanorods, which allows for further light illumination to pass through and excite more electrons. A sample prepared at the hydrothermal temperature of 120 °C was selected to proceed with the growth time parameter. Figure 8 shows the photocurrent density generated by the ZnO nanorods prepared at various growth times at the hydrothermal temperature of 120 °C. It was shown in Table 2 that the obtained photocurrent density showed a steady increment (from 0.1643 mA/cm^2^ to 0.1944 mA/cm^2^) as the growth time increased. The growth time was restricted to not more than 4 h because a longer growth time leads to thicker ZnO nanorods [34]. Consequently, this causes less efficient light harvesting and photocurrent conversion due to harder light penetration through the much thicker nanorods. Therefore, a hydrothermal temperature of 120 °C was the optimum temperature and a 4 h treatment was the selected best duration among the 1 to 4 h of hydrothermal time, due to the fact that an insignificant difference in current density value was recorded (0.0051 mA/cm^2^) between 3 h and 4 h of hydrothermal treatment. Since both samples displayed minute differences in terms of their generated current density, it was not advisable to extend the experimental works to a longer hydrothermal duration. However, there was the possibility that a higher photocurrent might have been generated by ZnO nanorods that underwent 5 h of hydrothermal treatment, considering the economic issue. It can be observed that the ZnO nanorods prepared at the best conditions (120 °C for 4 h) exhibited the highest photocurrent density of 0.1944 mA/cm^2^. This was supported by the generated photocurrent values (I_p_) of this particular sample prepared in various growth times by considering the dark current (I_d_) as shown in Figure 9. At +0.5 V, the actual current generated was found to be 0.1643 mA, 0.1871 mA, 0.1893 mA, and 0.1944 mA for 1 h, 2 h, 3 h and 4 h of growth time, respectively. Figure 9 shows an increasing trend in the current generated as the growth time increased. The sample prepared at 120 °C with the growth time of 4 h exhibited the highest current generated at the value of 0.1944 mA at the potential of +0.5 V.

These preparation conditions have enabled the ZnO seed layers to grow into highly ordered ZnO nanorods that may have provided photoinjected electrons with a direct electrical pathway to the photoanode and this has resulted in a fast charge transport in the photoelectrochemical system [6]. Different conductive substrates, like fluorine doped tin oxide glass (FTO) or titanium foil, as well as various electrolytes can be applied for further photoelectrochemical measurements.

## 4. Conclusions

Four hours of hydrothermal growth treatment at 120 °C has been proven to be the optimum condition to grow ZnO nanorods. Based on the FESEM micrograph, this particular sample exhibited less dense and non-closely packed nanorods, which allowed more penetration of light illumination in order to excite more electrons, ultimately resulting in a low band gap energy value of 3.13 eV and a higher current density when compared to other samples at the value of 0.1944 mA/cm^2^. Furthermore, it was found that a higher hydrothermal temperature contributes to a greater generation of current density. However, too high a hydrothermal temperature (130 °C) may cause vaporization of the Zn^2+^ source to occur as the boiling point of this precursor is 125 °C. This may have resulted in the inhibition of growth of the ZnO seeds and affected the structural, optical, and photoelectrochemical performance. Furthermore, the longer the growth time, the greater the photocurrent density observed. However, the growth time was restricted to not more than 4 h as a longer growth time leads to a thicker formation of ZnO nanorods and consequently causes less efficient light harvesting and photocurrent conversion. Moreover, extending the experimental works to a longer hydrothermal treatment duration was not advisable due to the insignificant difference in the current density value recorded between 3 h and 4 h of treatment (0.0051 mA/cm^2^), however, a sample prepared with 5 h of growth time might exhibit a greater photocurrent.

## Figures and Tables

**Table 1:** Photocurrent density of ZnO/ITO prepared at various hydrothermal temperatures.

ZnO/ITO Nanorods Prepared at Various Hydrothermal Temperatures (°C)	Photocurrent Density (mA/cm^2^)
90	0.1061
100	0.1686
110	0.1809
120	0.1944
130	0.0245
ITO	0.0028

**Table 2:** Photocurrent density of ZnO/ITO prepared at various growth time.

ZnO/ITO Nanorods Prepared at Various Growth Time (h)	Photocurrent Density (mA/cm^2^)
1	0.1643
2	0.1871
3	0.1893
4	0.1944
